# Non-Invasive Telemonitoring in Heart Failure: A Systematic Review

**DOI:** 10.3390/medicina61071277

**Published:** 2025-07-15

**Authors:** Patrick A. Kwaah, Emmanuel Olumuyide, Kassem Farhat, Barbara Malaga-Espinoza, Ahmed Abdullah, Michael H. Beasley, Novi Y. Sari, Lily K. Stern, Julio A. Lamprea-Montealegre, Adrian daSilva-deAbreu, Jiun-Ruey Hu

**Affiliations:** 1 Department of Internal Medicine, Yale School of Medicine, Waterbury, CT 06708, USA; 2 Department of Internal Medicine, Advocate Illinois Masonic Medical Centre, Chicago, IL 60657, USA; 3 Department of Internal Medicine, School of Medicine, University of Texas Rio Grande Valley, Edinburg, TX 78539, USA; 4 Department of Internal Medicine, Griffin Hospital, Derby, CT 06418, USA; 5 Section of Cardiovascular Medicine, Yale School of Medicine, New Haven, CT 06510, USA; 6 Siloam Heart Institute, Siloam Hospitals Kebon Jeruk, Jakarta 11530, Indonesia; 7 Department of Cardiology, Smidt Heart Institute, Cedars-Sinai Medical Center, Los Angeles, CA 90048, USA; 8 Division of Cardiology, Department of Medicine, University of California, San Francisco, CA 94143, USA; 9 Department of Cardiovascular Medicine, Mayo Clinic, Rochester, MN 55905, USA

**Keywords:** heart failure, telemonitoring, mortality, readmission, quality of life

## Abstract

*Background and Objectives:* Heart failure (HF) represents a major public health challenge worldwide, with rising prevalence, high morbidity and mortality rates, and substantial healthcare costs. Non-invasive telemonitoring has emerged as a promising adjunct in HF management, yet its clinical effectiveness remains unclear. *Materials and Methods:* In this systematic review, we summarize randomized controlled trials (RCTs) between 2004 and 2024 examining the efficacy of non-invasive telemonitoring on mortality, readmission, and quality of life (QoL) in HF. In addition, we characterize the heterogeneity of features of different telemonitoring interventions. *Results:* In total, 32 RCTs were included, comprising 13,294 participants. While some individual studies reported benefits, non-invasive telemonitoring demonstrated mixed effects on mortality, readmission rates, and QoL. The most common modality for interfacing with patients was by mobile application (53%), followed by web portals (22%), and stand-alone devices (19%). Periodic feedback (63%) was more common than continuous feedback (31%) or on-demand feedback (6%). Clinician reviews of patient telemonitoring data was event-triggered (44%) more commonly than based on a prespecified timeline (38%). In most designs (90%), patients played a passive role in telemonitoring. *Conclusions:* Non-invasive telemonitoring interventions for HF exhibited considerable variation in duration and system design and had a low rate of patient engagement. Future work should focus on identifying telemonitoring-responsive subgroups and refining telemonitoring strategies to complement traditional HF care.

## 1. Introduction

Heart failure (HF) remains a significant global health burden, associated with substantial morbidity and mortality [1,2]. In 2021, the global prevalence of HF was estimated to exceed 56 million cases [3]. In the United States (U.S.), one in four individuals will develop HF in their lifetime, and the prevalence is projected to rise to 8.5 million by 2030 [4]. The overall economic cost of HF was $108 billion in 2012 [5]. The human and economic costs of HF are projected to increase as the global population continues to expand, industrialize, and age [6]. Despite advances in preventive, pharmacologic, and device-based therapy, the prognosis of HF remains poor, with high mortality rates across all demographics, notably among younger adults and rural dwellers [4,7,8,9].

Telemonitoring in HF involves the remote use of technology to track patients’ clinical parameters and symptoms, aiming to optimize disease management, limit readmission, and reduce the cost of healthcare [10,11]. Telemonitoring can be classified into invasive and non-invasive methods. Invasive telemonitoring involves the use of implantable devices (such as pulmonary artery or vena cava pressure sensors and electrophysiological devices) that monitor hemodynamic and electrophysiological parameters, while non-invasive telemonitoring involves the use of external measurement of variables, such as weight, heart rate, blood pressure, or HF-related symptoms [10,12]. Although individual randomized controlled trials (RCTs) have shown inconsistent findings regarding home HF telemonitoring, recent meta-analyses have demonstrated an overall trend in the ability of both invasive and non-invasive telemonitoring to lead to improvements in mortality, readmissions, and quality of life (QoL) [10,11,13,14,15].

Given the more robust evidence for invasive telemonitoring and the relatively homogeneous interventions, we focus here on non-invasive telemonitoring, where the evidence is sparse, and the component interventions are more heterogeneous. Therefore, the primary aim of this systematic review is to provide updated estimates on the effect of RCTs on non-invasive HF telemonitoring on readmission, mortality, and QoL. The secondary aim of this systematic review is to dissect non-invasive telemonitoring interventions into their components.

## 2. Materials and Methods

This systematic review of RCTs was conducted using the Preferred Reporting Items for Systematic Reviews and Meta-Analyses (PRISMA) guidelines [16]. The review was registered on PROSPERO (protocol registration ID: CRD420250626040) and did not require ethics approval as it was a secondary analysis of published study-level data and did not include human subject interaction.

A comprehensive search of the PubMed database was conducted to identify relevant articles published in the last 20 years, from 1 January 2004 to 31 December 2024. The keywords used in the search strategy included: “heart failure”, “cardiac failure”, “telemedicine”, “telecare”, “virtual medicine”, “eHealth”, “mHealth”, “digital health”, “health application”, and “remote monitoring”. The full Boolean search strategy is provided in the Supplementary Material.

All retrieved articles were imported into Covidence for screening. Two individuals from our team of five independent reviewers (P.K., K.F., A.A., E.O., and B.M.E.) screened each title and abstract to identify potentially eligible studies and then analyzed full-text citations. In the event of a disagreement between two reviewers at any point, a third one resolved the conflict, and a final decision was reached by simple majority. A similar methodology of parallel review and management of disagreements was applied during each step listed below.

To warrant inclusion in this systematic review, studies had to meet all of the following criteria (Supplementary Material): (1) have only patients aged ≥ 18 years diagnosed with HF regardless of their New York Heart Association (NYHA) functional class, (2) involve home telemonitoring systems that were non-invasive, (3) be an RCT with a minimum sample size of 50 participants, (4) include a control group receiving usual treatment, with a clearly defined randomization process, (5) have its results published as full-text articles, (6) be published between 2004 and 2024, and (7) be published in the English language. We permitted the inclusion of studies of HF patients with or without coexisting conditions and studies involving a subset of the HF population (e.g., patients with left ventricular assist device [LVAD], atrial fibrillation [AF], specific combination with comorbid conditions). Articles were excluded if they met any of the following criteria: (1) conference abstracts, design protocols, systematic reviews, or meta-analyses, (2) studies conducted on patients who were in settings other than home, e.g., inpatient hospitals, rehabilitation centers, or nursing homes, (3) studies lacking the use of remote or wireless data transmission methods, (4) studies focusing solely on HF telerehabilitation, (5) interventions that were not explicitly designed to target HF, (6) eHealth or mHealth studies focusing solely on educational training without incorporating patient monitoring, (7) studies involving invasive (e.g., pulmonary arterial pressure sensors) HF telemonitoring techniques, or (8) studies focusing on algorithms for model development or others without original patient data.

The extracted data were entered into a pre-established Microsoft Excel sheet. The following variables were extracted from the main study reports (Table 1): First author, year, sample size, duration of the follow-up period, comorbidities, HF type, and NYHA class, guideline-directed medical therapy (GDMT), type and characteristics of telemonitoring parameters measured, outcomes, among other characteristics. If studies reported endpoints with multiple time points, data from the latest point were extracted.

**Figure 1:**
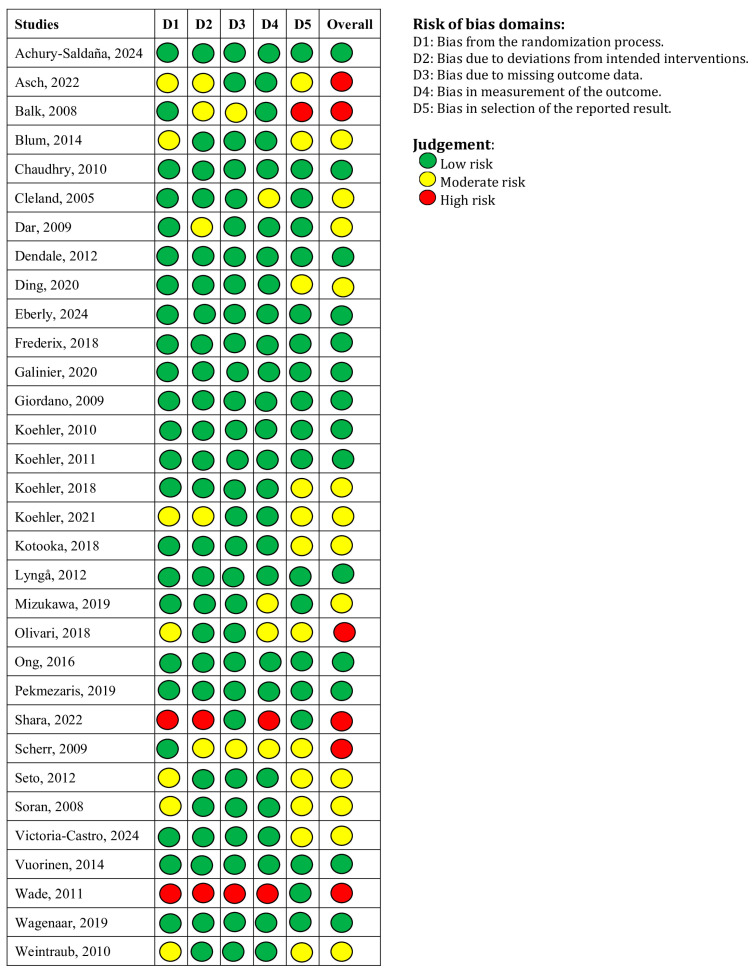
Risk of bias analysis of randomized controlled trials using RoB2 [18,19,20,21,22,23,24,25,26,27,28,29,30,31,32,33,34,35,36,37,38,39,40,41,42,43,44,45,46,47,48].

The risk of bias for RCTs was assessed using the Cochrane Risk of Bias 2 (RoB 2) tool [49]. Each study was evaluated across 5 domains: (1) bias due to the randomization process, (2) deviation from intended intervention, (3) missing outcome data, (4) measurement of outcomes, and (5) selection of the reported result. Each domain was judged as “low risk”, “high risk”, or “some concerns”, based on criteria defined by the Cochrane Collaboration. Each study’s overall risk of bias was determined according to the RoB 2 algorithm (Figure 1).

## 3. Results

### 3.1. Study Selection

**Figure 2:**
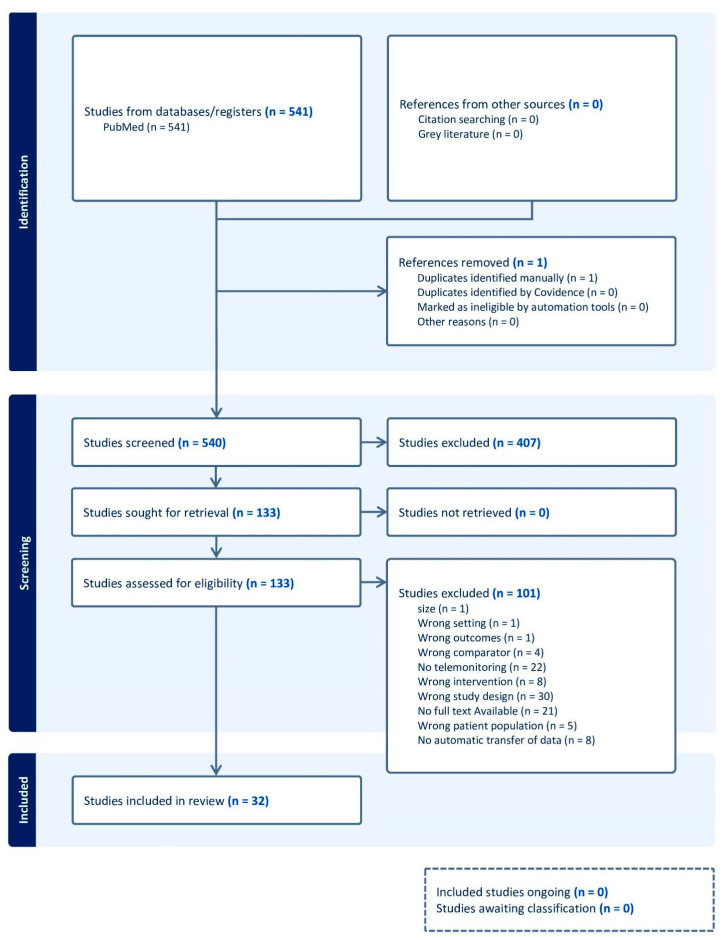
Preferred Reporting Items for Systematic Reviews and Meta-Analyses (PRISMA) flow-chart.

The search strategy identified 541 articles. One duplicate was removed, and the remaining articles were compiled for screening by title and abstract. Of these, 133 articles met the criteria for full-text review. After applying the aforementioned inclusion and exclusion criteria to the full-text review process, 32 manuscripts were ultimately included for analysis [17,18,19,20,21,22,23,24,25,26,27,28,29,31,32,33,34,35,36,37,38,39,40,41,42,43,44,45,46,47,48]. Information on the reasons for exclusion can be found in the PRISMA flow diagram shown in Figure 2.

### 3.2. Study Characteristics

A total of 32 randomized controlled trials encompassing 13,294 patients across 13 countries were included. The majority originated from the U.S. (35.0%), followed by Germany (15.0%), and the Netherlands (9.0%). Sample sizes ranged from 59 to 1653 participants, with mean ages between 55 and 80 years. Female representation varied from 15.0% to 65.0%. Racial demographics were predominantly reported in U.S.-based studies, with White participants comprising 41.0–86.0%, Black 8.0–78.0%, and Hispanic 1.0–8.0% (Supplementary Table S1). Hypertension was reported in 17 studies (21.0–81.0% prevalence), diabetes in 21 studies (25.0–49.0%), and ischemic heart disease in 14 studies (20.0–69.0%). Depression was reported infrequently (two studies; 10.0–20.0%). HF with reduced ejection fraction (HFrEF) was the most studied phenotype (14 trials), while HFmrEF and HFpEF were each reported in five studies. NYHA classes II and III predominated (reported in 19 and 20 studies, respectively; Table 1). Regarding pharmacologic therapy, RAAS inhibitors were reported in 23 trials (34.0–100% prevalence), with similar reporting rates for beta-blockers and MRAs (n = 23 each). SGLT2 inhibitor use was documented in only one trial. (Supplementary Table S2).

### 3.3. Features of Telemonitoring Interventions

There was significant heterogeneity in telemonitoring approaches. Each intervention per RCT was detailed in Table 2a. The definitions of all telemonitoring parameters in our review are included in the Supplementary Material. The most common modality for interfacing with patients was by mobile application (53.1%). Clinician interactions were predominantly reactive (59.4%), 25.0% were proactive, and 15.6% incorporated both approaches. The frequency of feedback for patients varied, with periodic feedback being the most frequent (62.5%). Among periodic feedback approaches, daily updates were the most common (59.0%), followed by weekly (12.5%). In most studies (90.6%), patients played a passive role in monitoring. Providers primarily reviewed patient data on an event-based basis (43.7%) or through routine reviews (37.5%), while 9.3% engaged in regular patient interactions.

Monitored data including hemodynamic parameters, symptoms, and laboratory assessments can be found in Table 2b. Follow-up durations varied among included studies, with 40.6% of studies reporting short-term follow-up (<6 months), 37.5% medium-term (6–12 months), and 21.8% long-term (>12 months) (Table 2b).

### 3.4. Outcomes of Telemonitoring Interventions

We summarized the outcomes assessed across all 32 RCTs. Regarding mortality, 17 studies examined all-cause mortality, five focused on cardiovascular-related mortality, and only two analyzed HF-related mortality. Regardless of follow-up duration, most studies did not demonstrate a mortality benefit with telemonitoring. Only Achury-Saldaña et al. 2024 reported a significant reduction in HF-related mortality in the telemonitored group (*p* = 0.024) [17] (Table 3a). Rehospitalization outcomes were more frequently assessed than mortality outcomes. Specifically, 19 studies evaluated all-cause rehospitalization, seven examined cardiovascular-related rehospitalization, and 19 focused on heart-failure-related rehospitalization. While most interventions showed no effect, 6 of 19 studies reported reduced HF-related rehospitalizations, with five conducted over 6 months (Table 3b). Lastly, eight studies evaluated QoL, with three demonstrating improvements, particularly in those with longer follow-up durations (≥12 months) (Table 3c). Several RCTs reported composite outcomes instead of individual outcomes, which we summarized in Supplementary Table S3.

None of the comparisons between intervention characteristics and key clinical outcomes of mortality, rehospitalization, and QoL were statistically significant after applying the Bonferroni correction for multiple comparisons. However, some associations did reach statistical significance before the Bonferroni correction, which we have made available in the Supplemental Results section.

## 4. Discussion

Our systematic review of 32 randomized control trials encompassing 13,294 patients with HF, across NYHA functional classes, who underwent non-invasive telemonitoring showed some benefits in some individual studies. However, the studies showed inconsistent results in the overall effect on heart failure readmission, all-cause mortality, cardiovascular mortality, and HF-related mortality. We also found a trend toward a neutral impact of telemonitoring on overall all-cause readmission, cardiovascular readmission, HF-related readmission, and QoL. This study provides an update to previous systematic reviews on non-invasive telemonitoring and represents the first study to characterize the component parts of HF telemonitoring interventions, to our knowledge.

Our study found that benefit of telemonitoring compared to usual care on rehospitalization rates was also variable in our review. Rehospitalization was a frequently assessed outcome, with 19 studies evaluating all-cause rehospitalization and HF rehospitalizations. Prior meta-analyses have reported conflicting results regarding the impact of telemonitoring on rehospitalization, likely due to variations in study designs, patient demographics, and healthcare access. While some studies reported a reduced risk of all-cause, cardiovascular, and HF-related readmissions [10,13,25,50,51,52], others found neutral effects on rehospitalization outcomes alone [39,53]. The effectiveness of telemonitoring may depend on the specific strategies implemented, particularly those incorporating structured patient feedback and clinician intervention. Findings from the TEMA-HF 1 trial suggest that, while telemonitoring can enhance clinical follow-up, particularly when integrated with HF clinics, it does not necessarily reduce hospitalization rates [24]. Our systematic review showed more passive patient and physician involvement, which likely contributed to the low effect seen. We categorized clinician interaction as proactive, reactive, or both. Although a pattern emerged suggesting a potential trend favoring proactive strategies, the limited number of studies restricts the ability to draw firm conclusions. Among the eight trials that employed proactive monitoring, five reported outcomes related to rehospitalization, with only two (40%) demonstrating a statistically significant reduction in heart failure-related rehospitalization. In contrast, of the 19 studies utilizing reactive monitoring, 16 reported rehospitalization outcomes, but only three showed significant findings. More interactive telemonitoring systems, which actively engage patients rather than relying on passive monitoring, have been associated with better adherence and improved clinical outcomes [54]. This suggests that telemonitoring alone may be insufficient in preventing readmissions unless combined with comprehensive in-person care and structured intervention strategies. Future research should focus on methodological improvements to better understand the variations in rehospitalization outcomes and determine the most effective telemonitoring strategies for different patient populations.

In addition, reported outcomes on mortality in telemonitoring had mixed effects on mortality compared to usual care. This finding aligns with the previous meta-analysis by Drews et al., which also reported no significant reduction in all-cause mortality with telemonitoring [53]. Among the 11 studies included in their analysis, the two largest ones reported a neutral effect, which was believed to have affected the overall results. Similarly, Zhu et al., in their systematic review, found no HF-related mortality benefit [50]. Among the 32 studies in our review, 17 assessed all-cause mortality, but only two reported a significant benefit of telemonitoring. Additionally, five studies evaluated cardiovascular mortality, with two showing a benefit, while only one specifically assessed HF-related mortality. A meta-analysis by Ding et al. observed a more significant mortality benefit with telemonitoring than usual care [25]. Their findings emphasized the importance of mobile health (mHealth) alongside telemonitoring, showing more significant benefit in experiences incorporating mHealth systems (0.67 [0.53–0.85]) compared to telemonitoring alone (0.95 [0.84–1.07]). In our systematic review, approximately 53% of the included RCTs utilized an mHealth interface alongside telemonitoring. The relatively small difference in frequency of mHealth utilization between studies that incorporated it and those that did not account for the smaller effect observed in our findings. Yun et al. also reported that telemonitoring reduced overall mortality; however, their subgroup analysis found no mortality benefit in short-term (<3 months) and medium-term (3–12 months) follow-ups, whereas long-term follow-ups (≥12 months) demonstrated a significant mortality reduction (0.81 [0.67–0.99]) [55]. In our systematic review, only 21.8% of follow-ups were long-term (>12 months), which may partially explain the limited mortality benefit observed. Furthermore, Scholte et al. found that structured telephone support, where HF patients received frequent calls from a nurse or cardiologist, significantly improved outcomes (0.75 [0.63–0.89]) compared to biometric data-based telemonitoring (0.95 [0.79–1.05]) [10]. Only 3 out of 32 RCTs in our systematic review included structured regular follow-ups, potentially contributing to these findings.

Regarding QoL, our study found that telemonitoring had mixed effects compared to usual care. Among the eight studies that reported QoL outcomes, three showed significant improvements, all of which had follow-ups of 12 months or longer. The remaining five studies, which did not find statistically significant improvements, included four with follow-ups of less than 12 months and one with a follow-up period exceeding 12 months. A study by Mizukawa et al. found a statistically significant improvement in QoL at 18 and 24 months among the telemonitoring group compared to usual care [36]. Similarly, a previous meta-analysis by Knox et al. reported a small but significant increase in overall QoL (SMD 0.23, 95% CI 0.09–0.37, *p* = 0.001) among patients undergoing telemonitoring. Further moderator analysis based on intervention duration revealed that shorter-duration interventions were not inferior to usual care, while longer-duration interventions (≥52 weeks) were associated with significant QoL improvements. Longer duration of telemonitoring has been associated with refined self-care skills sustained with constant engagement over time, reduced hospital and emergency admissions, and improved functional and health-related quality of life [56,57,58]. Improvements in QoL were more frequently reported in studies with longer follow-up durations (≥12 months), suggesting that sustained engagement in telemonitoring may be necessary to achieve meaningful patient-reported benefits in QoL. Del Toro et al., in their 2023 meta-analysis of 19 RCTs, reported significant variability in quality of life (QoL) outcomes, primarily due to inconsistencies in scoring systems and reporting measures, which hinder comprehensive assessment [59]. A critical review of the methodology of studies reporting QoL as an outcome revealed that periodic follow-up with questionnaires specifically assessing QoL prior to study completion was a common feature among those showing positive results (two out of three studies) [33,36]. In contrast, among the five studies reporting no improvement in QoL, only one employed frequent questionnaires during the study period. While we acknowledge that this does not constitute a definitive trend, these observations suggest a potential association worth further exploration [19]. Future RCTs should prioritize the standardization of QoL metrics to enable more consistent and meaningful comparisons across studies.

Although our systematic review yielded neutral findings overall, this should not be interpreted as justification to curtail the development or implementation of HF telemonitoring. On the contrary, the data highlights many successful telemonitoring interventions in improving clinical outcomes, so identifying targeted patient subgroups that would benefit the most should be identified in future studies, and tailored digital health interventions should be developed that fit the study groups. Identifying subpopulations to whom telemonitoring is ineffective is just as valuable, as this supports a greater emphasis on traditional clinic-based monitoring for these patients and allows a more efficient allocation of resources toward those most likely to benefit.

Our review also revealed substantial heterogeneity across studies concerning patient digital literacy and engagement, adherence to telemonitoring protocols and alert responses, and rates of “soft dropout” due to patient disengagement, all of which may have attenuated the observed effectiveness of interventions. Despite a notable increase in health-related internet use among American adults from 24.8% in 2009 to 43.9% in 2018 and the promise that telemonitoring holds for improving access to care among underserved rural populations, significant socioeconomic barriers persist [60,61]. This includes reduced health literacy and limited availability of broadband internet access that hinder the implementation of telemonitoring interventions and pose challenges to conducting research studies [60,61,62]. Furthermore, HF predominantly affects older individuals, who face additional challenges, including limited experience with modern technology, device-related complexities, health limitations from comorbidities, and potential lack of social or family support [63]. These digital and structural disparities raise an important ethical imperative. Without intentional, equity-focused design, telemonitoring innovations risk exacerbating disparities in HF outcomes by excluding the very populations most vulnerable to disease progression. Frameworks such as the Digital Health Equity Framework should be integrated into both trial design and clinical implementation to ensure telemonitoring tools are usable, accessible, and responsive to the needs of older adults, socioeconomically disadvantaged individuals, rural dwellers, and racially or ethnically minoritized populations. [64]. Importantly intersecting social identities, such as age, race, gender, disability, and income, may compound challenges to engagement, underscoring the need for disaggregated data reporting to uncover hidden disparities [65]. Language and cultural barriers also restrict access, as many platforms are not available in languages other than English and lack culturally adaptive design [66]. Additionally, caregivers play a crucial role in helping older adults use digital tools, yet they are rarely incorporated into intervention protocols, potentially limiting real-world adoption. Policy and reimbursement structures further influence access, as many telemonitoring programs are not fully reimbursed for Medicaid beneficiaries or uninsured populations, which can reinforce inequities in care delivery [67]. To promote health equity in digital HF care, future research should incorporate community-partnered approaches, human-centered design, and universal usability principles. Trials should also report equity and inclusion metrics—such as digital literacy screening, language accessibility, and caregiver involvement—to ensure that telemonitoring technologies do not leave vulnerable populations behind [68].

Our review revealed substantial heterogeneity of types of clinician interaction and patient engagement methods. The literature has emphasized the importance of proactive management strategies in promoting effective remote patient monitoring (RPM), as demonstrated by the Trans-European Network-Home-Care Management System (TEN-HMS) study and the Telemedical Interventional Management in HF II (TIM-HF2) trial [22,32]. Clinically, this approach enables early intervention during the pre-symptomatic phase of HF decompensation by identifying patients who may benefit from medication up-titration and facilitating timely adjustments to clinical workflows instead of relying solely on reactive strategies [69]. However, it is unclear if proactive monitoring alone consistently translates into improved primary outcomes across these studies. This underscores the need for further investigation and refinement of the telemonitoring applications within a well-characterized subgroup of HF patients. Concerning patients’ involvement, most trials (90.6%) employed a passive monitoring model. Although several trials characterized by active patients’ engagement, such as Chaudhry et al., failed to demonstrate a benefit over usual care, other studies, including SPAN-CHF II and TIM-HF2, reported significant improvement in clinical outcomes [22,32,48,69]. The aforementioned finding suggests that active patient engagement may be associated with better outcomes; however, the observed heterogeneity may be related to the influence of engagement levels and the complexity of the telemonitoring system. Given the substantial clinical and methodological heterogeneity across interventions—including variations in monitoring technologies, clinician responsiveness, and patient engagement—we determined that a meta-analytic approach was not appropriate. Consequently, no pooled estimates were generated, and statistical measures of heterogeneity (e.g., I^2^) were not calculated. Combining such diverse interventions into a single summary effect would risk oversimplification and misinterpretation of the data.

We also observed considerable variation in the types of telemonitoring platforms employed and the frequency of telemonitoring feedback delivered. Web-based platforms were the most used technological modality across the included studies. Prior meta-analyses have reported conflicting findings regarding the relative effectiveness of mobile- and web-based platforms. Some studies suggest that these platforms offer greater accessibility and enhance patient engagement compared to traditional phone- or device-based monitoring [52,55,59]. However, the effectiveness of telemonitoring may also be influenced by contextual factors, such as digital literacy, provider responsiveness, and integration with routine clinical workflows [55,70]. Patient engagement emerged as a critical yet underutilized component across the telemonitoring interventions reviewed. Only approximately 9.3% of studies employed models that required active participation from patients. The overwhelming majority relied on passive data collection, with minimal patient interaction. This low level of engagement rate likely limited the effectiveness of telemonitoring in improving clinical outcomes, as active involvement is increasingly recognized as essential for enhancing adherence, self-management, and clinical benefit [52,54]. Regarding feedback frequency, 62.5% of studies implemented periodic feedback, most commonly daily (59%), while 31.2% used continuous monitoring, and 6.2% provided on-demand feedback. Frequent telemonitoring has been shown to detect early symptoms, prompt intervention, and reduce mortality and hospitalization. Previous systematic reviews have noted that frequent patient feedback can improve adherence to self-care recommendations [55]. In contrast, studies reporting neutral outcomes, such as Umeh et al., suggest that although frequent monitoring may not reduce event rates, it can facilitate earlier recognition of decompensation, potentially leading to shorter hospital stays due to earlier and less severe presentations [11]. While these findings support the potential benefits of frequent monitoring, there is concern that such systems may contribute to alarm fatigue and increased healthcare utilization, with uncertain impact on long-term outcomes such as mortality and readmission rates. Lastly, regarding system integration, only a minority (12.5%) of platforms were integrated into the EHR, reflecting the disjointed digital health ecosystem and the need to develop systems to streamline communication between digital health tools.

### Limitations

Our systematic review is subject to several important limitations. First, many studies included in the analysis had incomplete reports of their methodologies, making it difficult to assess their risk of bias despite our use of the Risk of Bias 2.0 algorithm. Descriptive details were lacking in specific components of telemonitoring interventions, underscoring the underlying issue that telemonitoring is too often treated as a monolithic single entity. We did not explicitly exclude RCTs with a high risk of bias, although we excluded those with small sample sizes of <50 participants. Second, variations in clinic-based standard care in the non-telemonitoring arm and differential rates in drop-out and patient digital literacy may have diluted the impact of telemonitoring on outcomes. One could expect such technological literacy to have improved over time. Third, the care of HF patients has significantly changed over these past two decades and, likely, the clinical course of patients from the early or mid-2000s differed significantly from what we observe in current clinical practice, not just due to the adoption of angiotensin receptor and neprilysin inhibition, SGLT inhibitors, and to some extent, intravenous iron in HFrEF, but also the emphasis on maximizing all GDMT agents, even with the use of agents to treat GDMT-induced hyperkalemia if needed. Fourth, without pooled effect estimates, the ability to draw definitive conclusions about the efficacy of telemonitoring across different populations and clinical settings is constrained. However, in light of the heterogeneity in the above factors, it is not clear that performing a meta-analysis of the component features of telemonitoring interventions at the present time would be meaningful, but this should remain a consideration as the field of HF telemonitoring interventions evolves.

## 5. Conclusions

Non-invasive telemonitoring in heart failure shows variable effects on clinical outcomes, with benefits emerging in certain contexts. This variability often reflects differences in patient engagement, duration of follow-up, clinician response, and system integration. Although the overall evidence remains mixed, clinicians might cautiously favor mHealth solutions, given their flexibility and potential to enhance patient engagement, especially among digitally literate individuals. Proactive monitoring approaches may be advantageous for high-risk patients and deserve consideration over purely reactive strategies. Actively involving patients is key to improving adherence and outcomes, and longer monitoring periods of at least 12 months may be necessary to achieve meaningful gains in quality of life. Clinicians should tailor telemonitoring to individual patient needs, digital literacy, and social factors to avoid widening health disparities. Future research should refine interventions, identify responsive patient subgroups, and integrate telemonitoring into proactive care models to maximize its benefits.

## Figures and Tables

**Table 1:** Baseline characteristics and comorbidities in the included studies.

First Author, Year and Total Population (*N*)	HFrEF (EF < 40%)	HFmrEF (EF 41–49%)	HFpEF (EF ≥ 50%)	Diabetes (%)	Hypertension (%)	Smoking (%)	Dyslipidemia/ Hyperlipidemia (%)	CKD (%)	COPD (%)	Ischemic Heart Disease /CAD (%)	Depression
Achury-Saldaña 2024 ^a^ (*N* = 140) [17]	N/A	N/A	N/A	I: 25.7 C: 30.0 T: 27.9	N/A	N/A	I: 50.0 C: 34.3 T: 42.1	I: 42.9 C: 32.9 T: 37.9	I: 11.4 C: 14.3 T: 12.9	N/A	N/A
Asch 2022 ^a^ (*N* = 552) [18]	N/A	N/A	N/A	I: 45.30 C: 53.2 T: 49.3	I: 49.8 C: 48.8 T: 49.3	N/A	N/A	I: 38.9 C: 35.6 T: 37.2	N/A	N/A	N/A
Balk 2008 (*N* = 214) [19]	I: 31.0 C: 31.0 T: 31.0	N/A	N/A	I: 30.0 C: 31.0 T: 30.5	I: 35.0 C: 30.0 T: 32.5	N/A	N/A	N/A	I: 27.0 C: 20.0 T: 23.5	I: 53.0 C: 61.0 T: 57.0	N/A
Blum 2014 ^a^ (*N* = 206) [20]	N/A	N/A	N/A	N/A	N/A	N/A	N/A	N/A	N/A	N/A	N/A
Chaudhry 2010 (*N* = 1653) [21]	I: 71.0 C: 70.2 T: 70.6	N/A	N/A	I: 47.7 C: 45.7 T: 46.7	I: 76.5 C: 77.3 T: 76.9	N/A	N/A	I: 54.6 C: 53.5 T: 54.0	I: 20.5 C: 21.4 T: 20.9	I: 52.3 C: 48.7 T: 50.5	N/A
Cleland 2005 ^a^ (*N* = 426) [22]	N/A	N/A	N/A	I: 35.0 C: 35.0 T: 35.0	I: 44.0 C: 40.0 T: 45.66	N/A	N/A	N/A	I: 24.0 C: 29.0 T: 25.0	I: 61.0 C: 68.0	N/A
Dar 2009 ^a^ (*N* = 182) [23]	N/A	N/A	N/A	I: 34.0 C: 37.0 T: 35.7	I: 60.0 C: 63.0 T: 61.5	N/A	N/A	I: 69.0 C: 69.0 T: 69.0	I: 9.0 C: 9.0 T: 9.0	N/A	N/A
Dendale 2012 ^a^ (*N* = 160) [24]	N/A	N/A	N/A	N/A	N/A	N/A	N/A	N/A	N/A	N/A	N/A
Ding 2020 (*N* = 184) [25]	I: 7.1 C: 15.9 T: 11.5	N/A	N/A	I: 32.0 C: 42.0 T: 37.0	N/A	N/A	N/A	I: 11.0 C: 22.0 T: 16.5	I: 25.0 C: 22.0 T: 23.5	N/A	N/A
Eberly 2024 ^a^ (*N* = 103) [26]	N/A	N/A	N/A	N/A	N/A	N/A	N/A	N/A	N/A	N/A	N/A
Frederix 2018 (*N* = 160) [27]	I: 35.0 C: 37.0 T: 36.0	N/A	N/A	N/A	N/A	N/A	N/A	I: 11.0 C: 11.0 T: 11.0	N/A	N/A	N/A
Galinier 2020 (*N* = 900) [28]	I: 58.5 C: 61.5 T: 60.0	I: 19.8 C: 19.6 T: 19.7	I: 21.7 C: 18.9 T: 20.3	I: 30.3 C: 35.4 T: 32.8	I: 61.4 C: 54.1 T: 57.8	I: 23.0 C: 25.5 T: 24.2	I: 55.8 C: 54.5 T: 55.2	I: 26.8 C: 28.1 T: 27.4	I: 18.5 C: 19.6 T: 19.0	I: 49.0 C: 50.1 T: 49.5	N/A
Giordano 2007 ^a^ (*N* = 460) [29]	N/A	N/A	N/A	I: 29.0 C: 27.0 T: 28.0	I: 19.0 C: 23.0 T: 21.0	N/A	N/A	N/A	I: 28.0 C: 26.0 T: 27.0	I: 53.0 C: 51.0 T: 52.0	N/A
Koehler 2010 (*N* = 710) [30]	I: 60.0 C: 59.0 T: N/A	N/A	I: 40.0 C: 41.0 T: N/A	I: 30.5 C: 29.5 T: N/A	I: 72.0 C: 72.0 T: N/A	I: 56.0 C: 55.0 T: N/A	I: 64.0 C: 63.0 T: N/A	I: 20.0 C: 19.0 T: N/A	I: 14.0 C: 13.0 T: N/A	I: 54.0 C: 53.0 T: N/A	N/A
Koehler 2011 (*N* = 710) [31]	N/A	N/A	N/A	I: 39.8 C: 39.3 T: 39.6	I: 68.0 C: 66.0 T: 67.0	N/A	I: 74.0 C: 74.7 T: 74.4	N/A	N/A	I: 57.1 C: 54.5 T: 55.8	N/A
Koehler 2018 (*N* = 1571) [32]	I: 45.0 C: 42.0 T: 43.6	I: 30.0 C: 35.0 T: 32.5	I: 25.0 C: 22.0 T: 23.9	I: 45.0 C: 46.0 T: 45.6	I: 17.0 C: 19.0 T: 18.8	I: 10.0 C: 7.0 T: 8.7	I: 55.0 C: 54.0 T: 54.2	N/A	N/A	I: 39.0 C: 42.0 T: 40.6	N/A
Koehler 2021 ^a^ (*N* = 674) [33]	N/A	N/A	N/A	N/A	N/A	N/A	N/A	N/A	N/A	N/A	N/A
Kotooka 2018 ^a^ (*N* = 181) [34]	N/A	N/A	N/A	N/A	N/A	N/A	N/A	N/A	N/A	I: 31.1 C: 29.7 T: 30.4	N/A
Lyngå 2012 (*N* = 344) [35]	I: 81.9 C: 78.4 T: 80.2	I: 18.1 C: 21.6 T: 19.7	N/A	I: 24.1 C: 28.8 T: 26.3	N/A	I: 8.4 C: 6.5 T: 7.5	N/A	N/A	N/A	I: 47.0 C: 45.1 T: 46.1	N/A
Mizukawa 2019 (*N* = 59) [36]	N/A	N/A	N/A	I: 36.8 C: 45.0 T: 42.4	I: 70.0 C: 63.2 T: 59.3	N/A	I: 60.0 C: 63.2 T: 57.6	N/A	N/A	N/A	N/A
Olivari 2018 ^a^ (*N* = 339) [37]	N/A	N/A	N/A	I: 38.9 C: 26.4 T: 32.5	N/A	N/A	N/A	I: 29.7 C: 31.8 T: 30.75	I: 19.7 C: 19.1 T: 19.4	N/A	N/A
Ong 2016 (*N* = 1437) [38]	N/A	I: 42.7 C: 43.0 T: N/A	N/A	I: 44.8 C: 47.6 T: N/A	I: 81.7 C: 80.1 T: N/A			I: 39.0 C: 42.7 T: N/A	I: 32.4 C: 32.5 T: N/A	N/A	I: 10.6 C: 11.1 T: N/A
Pekmezaris 2019 (*N* = 104) [39]	I: 58.0 C: 63.0 T: 61.0	I: 9.0 C: 11.0 T: 10.0	I: 33.0 C: 26.0 T: 29.0	N/A	N/A	N/A	N/A	N/A	N/A	N/A	N/A
Shara 2022 ^a^ (*N* = 60) [40]	N/A	N/A	N/A	N/A	N/A	N/A	N/A	N/A	N/A	N/A	N/A
Scherr 2009 (*N* = 120) [41]	I: 100.0 C: 100.0 T: 100.0	N/A	N/A	I: 30.0 C: 22.0 T: 25.9	I: 44.0 C: 54.0 T: 49.1	N/A	N/A	N/A	N/A	I: 43.0 C: 37.0 T: 39.8	N/A
Seto 2012 ^a^ (*N* = 100) [42]	N/A	N/A	N/A	N/A	N/A	N/A	N/A	N/A	N/A	I: 20.0 C: 26.0	N/A
Soran 2008 ^a^ (*N* = 315) [43]	N/A	N/A	N/A	N/A	N/A	N/A	N/A	N/A	N/A	N/A	N/A
Victoria-Castro 2024 (*N* = 182) [44]	I1: 71.7 I2: 63.0 I3: 65.2 C: 81.8 T: 70.3	N/A	I1: 28.3 I2: 37.0 I3: 34.8 C: 18.2 T: 29.7	I1: 45.7 I2: 41.3 I3: 41.3 C: 38.5 T: 41.8	I1: 76.1 I2: 78.3 I3: 80.4 C: 52.3 T: 72.0	N/A	I1: 37.0 I2: 26.1 I3: 45.7 C: 40.9 T: 37.4	I1: 26.1 I2: 23.9 I3: 19.6 C: 13.6 T: 20.9	I1: 21.7 I2: 15.2 I3: 19.6 C: 9.1 T: 16.5	I1: 45.7 I2: 28.3 I3: 28.3 C: 25.0 T: 31.9	I1: 30.0 I2: 15.2 I3: 17.4 C: 15.9 T: 19.8
Vuorinen 2014 (*N* = 94) [45]	I: 27.3 C: 28.6 T: 28.0	N/A	N/A	I: 4.0 C: 4.0 T: 4.0	I: 17.0 C: 13.0 T: 15.0	I: 76.0 C: 89.0 T: 82.5	N/A	I: 2.0 C: 9.0 T: 5.5	I: 4.0 C: 11.0 T: 7.5	N/A	N/A
Wade 2011 ^a^ (*N* = 316) [46]	N/A	N/A	N/A	N/A	N/A	N/A	N/A	N/A	N/A	N/A	N/A
Wagenaar 2019 (*N* = 450) [47]	I1: 73.3 I2: 66.7 C: 71.3 T: N/A	N/A	N/A	I1: 36.0 I2: 40.0 C: 39.0 T: N/A	I1: 41.3 I2: 43.3 C: 46.7 T: N/A	I: 12.0 I2: 14.0 C: 19.3 T: N/A	I1: 34.7 I2: 34.0 C: 28.7 T: N/A	I1: 15.3 I2: 16.0 C: 14.7 T: N/A	I1: 29.3 I2: 24.0 C: 20.0 T: N/A	I1: 39.3 I2: 48.0 C: 47.3 T: N/A	N/A
Weintraub 2010 (*N* = 188) [48]	I: N/A T: 30.0	N/A	N/A	I: 47.4 C: 38.7 T: 43.0	I: 44.2 C: 57.2 T: 69.0	N/A	N/A	N/A	N/A	I: 85.3 C: 87.1	N/A

Note: ^a^ Studies that reported ejection fraction only as thresholds or used non-standard classifications were reported as N/A:. I, intervention; C, control; CKD, chronic kidney disease; COPD, chronic obstructive pulmonary disease; HFmrEF, heart failure with mildly reduced ejection fraction; HFpEF, heart failure with preserved ejection fraction; HFrEF, heart failure with reduced ejection fraction; T, total; N/A, not available.

**Table 2:** (**a**) Telemonitoring characteristics of included studies. (**b**) Telemonitoring characteristics of included studies.

(**a**)						
**First Author** **and Year**	**Patient Interface**	**Clinician Interaction**	**Feedback** **Mechanism**	**Data Transmission**	**Patient** **Role**	**Provider** **Role**
Achury-Saldaña 2024 [17]	Mobile device/ app	Reactive	Continuous	Device-to-provider direct	Passive data collection	Event-based intervention
Asch 2022 [18]	Standalone monitoring devices	Reactive	On-demand	Device-to-provider direct	Passive data collection	Event-based intervention
Balk 2008 [19]	Web portal	Proactive	Periodic—Daily	Cloud-based	Passive data collection	Routine review of data
Blum 2014 [20]	Web portal	Reactive	Continuous	Device-to-provider direct	Passive data collection	Both event-based and regular
Chaudhry 2010 [21]	Phone call	Reactive	Periodic—Daily	Cloud-based	Passive data collection	Routine review of data
Cleland 2005 [22]	Standalone monitoring devices	Reactive	Periodic—Daily	Device-to-provider direct	Passive data collection	Event-based intervention
Dar 2009 [23]	Mobile device/app	Reactive	Continuous	Cloud-based	Passive data collection	Routine review of data
Dendale 2012 [24]	Mobile device/app	Reactive	Continuous	Device-to-provider direct	Passive data collection	Both event-based and regular
Ding 2020 [25]	Mobile device/app	Reactive	Periodic—Daily	Cloud-based	Passive data collection	Event-based intervention
Eberly 2024 [26]	Phone call	Reactive	Continuous	Cloud-based	Passive data collection	Routine review of data
Frederix 2018 [27]	Web portal	Reactive	Periodic—Daily	Cloud-based	Passive data collection	Event-based intervention
Galinier 2020 [28]	Mobile device/app	Reactive	Periodic—Daily	Cloud-based	Passive data collection	Event-based intervention
Giordano 2007 [29]	Mobile device/app	Both	Periodic—Weekly	Device-to-provider direct	Passive data collection	Regular interaction with the patient
Koehler 2010 [30]	Mobile device/app	Proactive	Continuous	Device-to-provider direct	Passive data collection	Regular interaction with the patient
Koehler 2011 [31]	Mobile device/app	Proactive	On-demand	Cloud-Based	Passive data collection	
Koehler 2018 [32]	Mobile device/app	Proactive	Periodic—Daily	Cloud-Based	Passive data collection	Routine review of data
Koehler 2021 [33]	Mobile device/app	Proactive	Periodic—Monthly	Device-to-provider direct	Passive data collection	Regular interaction with the patient
Kotooka 2018 [34]	Standalone monitoring devices	Reactive	Periodic—Daily	Cloud-based	Passive data collection	Routine review of data
Lyngå 2012 [35]	Web portal	Reactive	Periodic—Daily	Cloud-based	Active engagement	Routine review of data
Mizukawa 2019 [36]	Standalone monitoring devices	Both	Continuous	Cloud-based	Passive data collection	Event-based intervention
Olivari 2018 [37]	Standalone monitoring devices	Reactive	Periodic—Daily	Cloud-based	Passive data collection	Routine review of data
Ong 2016 [38]	Mobile device/app	Reactive	Periodic—Daily	Cloud-based	Passive data collection	Event-based intervention
Pekmezaris 2019 [39]	Mobile device/app	Proactive	Periodic—Daily	Cloud-based	Passive data collection	Routine review of data
Shara 2022 [40]	Mobile device/app	Proactive	Periodic—Daily	Cloud-based	Passive data collection	Routine review of data
Scherr 2009 [41]	Web portal	Both	Periodic—Daily	Cloud-based	Passive data collection	Event-based intervention
Seto 2012 [42]	Mobile device/app	Reactive	Continuous	Cloud-based	Active engagement	Event-based intervention
Soran 2008 [43]	Standalone monitoring devices	Reactive	Periodic—Daily	Device-to-provider direct	Passive data collection	Routine review of data
Victoria-Castro 2024 [44]	Mobile device/app	Both	Periodic—Daily	Cloud-based	Passive data collection	Routine review of data
Vuorinen 2014 [45]	Mobile device/app	Reactive	Periodic—Weekly	Cloud-based	Passive data collection	Event-based intervention
Wade 2011 [46]	Web portal	Reactive	Continuous	Cloud-based	Passive data collection	Event-based intervention
Wagenaar 2019 [47]	Web portal	Both	Continuous	Stored locally and uploaded periodically	Active engagement	Event-based intervention
Weintraub 2010 [48]	Mobile device/app	Proactive	Periodic—Daily	Cloud-based	Passive data collection	Routine review of data
(**b**)						
**First Author** **and Year**	**Hemodynamics** **Variables Monitored**	**Symptomatic** **Variables** **Monitored**	**Behavioral** **Variables** **Monitored**	**Laboratory** **Variables** **Monitored**	**Follow Up**	**Integration with Healthcare Systems**
Achury-Saldaña 2024 [17]	Weight, BP, HR	Dyspnea, Fatigue Ankle swelling dizziness	N/A	N/A	Medium-term	Standalone system
Asch 2022 [18]	Weight	N/A	Medication adherence	N/A	Medium-term	Linked to EHRs
Balk 2008 [19]	Weight, BP	N/A	N/A	N/A	Short-term	Standalone system
Blum 2014 [20]	Weight, BP HR, EKG	N/A	Medication adherence	Pro BNP	Medium-term	Standalone system
Chaudhry 2010 [21]	Weight	Dyspnea, Ankle swelling, depression	N/A	N/A	Medium-term	Community care integration
Cleland 2005 [22]	Weight, BP, HR, EKG	Dyspnea, Fatigue, Cough, Ankle swelling	N/A	RFT, Sodium	Long-term	Standalone system
Dar 2009 [23]	BP, HR, Weight, Pulse Oximetry	Dyspnea, Orthopnea, Ankle swelling, dizziness	N/A	N/A	Medium-term	Standalone system
Dendale 2012 [24]	Weight, BP, HR	N/A	Medication Adherence	N/A	Short-term	Community care integration
Ding 2020 [25]	Weight	N/A	N/A	N/A	Short-term	Standalone system
Eberly 2024 [26]	Weight, BP, HR	N/A	Medication Adherence	RFT	Short-term	Linked to EHRs
Frederix 2018 [27]	Weight, BP, HR	N/A	N/A	N/A	Medium-term	Standalone system
Galinier 2020 [28]	Weight	Dyspnea, Fatigue, Orthopnea, Ankle swelling, Cough	Medication Adherence	N/A	Long-term	Standalone system
Giordano 2007 [29]	Weight, BP	Fatigue, Dyspnea	N/A	N/A	Medium-term	Community care integration
Koehler 2010 [30]	Weight, BP, HR	N/A	Medication Adherence	N/A	Short-term	Linked to EHRs
Koehler 2011 [31]	Weight, BP, HR	N/A	N/A	N/A	Long-term	Linked to EHRs
Koehler 2018 [32]	Weight, BP, HR, EKG, Pulse Oximetry	N/A	N/A	Pro BNP	Long-term	Standalone system
Koehler 2021 [33]	Weight, BP, HR	N/A	N/A	Pro BNP	Medium-term	Standalone system
Kotooka 2018 [34]	Weight, BP, HR	N/A	N/A	Pro BNP, LVEF Change	Long-term	Community care integration
Lyngå 2012 [35]	Weight	Fatigue, Dyspnea, Ankle Swelling	N/A	N/A	Short-term	Standalone system
Mizukawa 2019 [36]	Weight, BP, HR	N/A	N/A	N/A	Long-term	Standalone system
Olivari 2018 [37]	Weight, BP, HR, EKG, Pulse Oximetry	N/A	N/A	N/A	Medium-term	Standalone system
Ong 2016 [38]	Weight, BP, HR	N/A	N/A	N/A	Short-term	Standalone system
Pekmezaris 2019 [39]	Weight, BP, HR, Pulse Oximetry	N/A	N/A	N/A	Short-term	Standalone system
Shara 2022 [40]	Weight	Dyspnea, Cough, Ankle swelling	Medication Adherence	N/A	Short-term	Standalone system
Scherr 2009 [41]	Weight, BP, HR	N/A	Medication Adherence	N/A	Medium-term	Standalone system
Seto 2012 [42]	BP, HR, EKG	Dyspnea, Depression	Physical Activity	Pro BNP	Short-term	Standalone system
Soran 2008 [43]	Weight	Dyspnea, Orthopnea, Fatigue, Ankle swelling	N/A	N/A	Medium-term	Community care integration
Victoria-Castro 2024 [44]	Weight, BP, HR	Fatigue, Weakness	Medication Adherence	N/A	Short-term	Standalone system
Vuorinen 2014 [45]	Weight, BP	Dyspnea, Fatigue, Ankle swelling, Palpitation	N/A	N/A	Medium-term	Standalone system
Wade 2011 [46]	Weight, BP	N/A	Medication Adherence	N/A	Short-term	Standalone system
Wagenaar 2019 [47]	Weight, BP, HR	N/A	Medication Adherence	N/A	Long-term	Standalone system
Weintraub 2010 [48]	Weight, BP, HR	Weakness, Fatigue	Medication Adherence	N/A	Short-term	Standalone system

(**a**) Note: N/A, Not available; BP, Blood pressure; HR, Heart rate; EKG, Electrocardiogram; RFT, Renal function test; EHR, Electronic health records; ProBNP, Pro-B-type natriuretic peptide; LVEF, Left ventricular ejection fraction. (**b**) BP: blood pressure; EHR: electronic health records; EKG: electrocardiogram; HR: heart rate; LVEF: left ventricular ejection fraction; N/A: not available; ProBNP: pro-b-type natriuretic peptide; RFT: renal function test.

**Table 3:** (**a**) Summary of follow-up duration and mortality outcomes in included RCTs. (**b**) Summary of follow-up duration and rehospitalization outcomes in included RCTs. (**c**) Summary of quality of life instruments, follow-up duration, and outcomes in included RCTs.

(**a**)				
	**Follow Up Period**	**Mortality**		
		**All-Cause**	**Cardiovascular**	**Heart Failure**
Achury-Saldaña 2024 [17]	6 months	N/A	N/A	I: 4.3%; C: 15.7% *p* = 0.024
Blum 2014 [20]	12 months	RR: 1.11 (0.71–1.73) *p* = 0.575	N/A	N/A
Chaudhry 2010 ^a^ [21]	6 months	HR: 0.97 (0.73–1.30) *p* = 0.86	N/A	N/A
Cleland 2005 [22]	450 days	I: 34%; C: 51% *p* value not analyzed	N/A	N/A
Dar 2009 ^b^ [23]	6 months	I: 178; C: 180 *p* = 0.3	N/A	N/A
Dendale 2012 ^c^ [24]	6 months	I: 5.0%; C: 17.5% *p* = 0.012	N/A	N/A
Frederix 2018 [27]	79 months	HR: 0.83 (0.57–1.20) *p* = 0.32	N/A	N/A
Giordano 2007 [29]	12 months	I: 9%; C: 14% *p* value not analyzed	RR: 0.44 (0.20–0.97) *p* = 0.04	N/A
Koehler 2010 [30]	24 months	RR: 0.95 (0.67–1.34) *p* = 0.76	N/A	N/A
Koehler 2011 [31]	26 months	HR: 0.97 (0.67–1.41) *p* = 0.87	HR: 0.86 (0.56–1.31) *p* = 0.49	N/A
Koehler 2018 [32]	393 days	HR: 0.70 (0·50–0·96) *p* = 0.0280	HR: 0·67 (0·45–1·01) *p* = 0.0560	N/A
Kotooka 2018 ^a^ [34]	12 months	HR: (0.354–1.847) *p* = 0.614	HR: (0.176–1.557) *p* = 0.245	N/A
Lyngå 2012 [35]	12 months	HR: 0.9 (0.65–1.26) *p* = 0.54	N/A	N/A
Mizukawa 2019 ^a^ [36]	24 months	I: 15.0%; C: 15.8% *p* = 0.859	N/A	N/A
Olivari 2018 ^a^ [37]	12 months	RR: 1.1 (0.72–1.68) *p* = 0.097	N/A	N/A
Ong 2016 [38]	6 months	I: 14%; C: 15.8% *p* = 0.30	N/A	N/A
Seto 2012 [42]	6 months	HR: 1.11 (0.62–1.99) *p* = 0.575	N/A	N/A
Soran 2008 ^a^ [43]	6 months	HR: 0.7 (0.32–1.52) *p* = 0.37	HR: 0.56 (0.2–1.55) *p* = 0.27	N/A
Wade 2011 [46]	6 months	RR: 1.08 (0.83–1.40) *p* = 0.575	N/A	N/A
Wagenaar 2019 ^d^ [47]	12 months	I1: HR: 2.82 (0.90–8.87) I2: HR: 2.06 (0.62–6.84)	N/A	I1: HR: 2.39 (0.62–9.24) I2: HR: 1.03 (0.21–5.11)
(**b**)				
	**Follow Up Period**	**Readmission/Rehospitalization**		
		**All-Cause**	**Cardiovascular**	**Heart Failure**
Achury-Saldaña 2024 [17]	6 months	N/A	N/A	I: 17.6%; C: 82.4% *p* = 0.008
Balk 2008 ^a^ [19]	537 days	I: 103 (0–14); C: 96 (0–7) *p* value not analyzed	I: 52%; C: 52% *p* value not analyzed	I: 19%; C: 19% *p* value not analyzed
Blum 2014 [20]	12 months	RR: 1.04 (0.87–1.25) *p* = 0.51	N/A	N/A
Chaudhry 2010 ^b^ [21]	6 months	HR: 1.06 (0.93–1.22) *p* = 0.39	N/A	N/A
Dar 2009 ^c^ [23]	6 months	N/A	N/A	I: 36%; C: 81% *p* = 0.01
Dendale 2012 ^d^ [24]	6 months	I: 0.80 ± 0.97 C: 0.82 ± 0.93 *p* = 0.93	N/A	I: 0.24 + 0.51 C: 0.42 + 0.70 *p* = 0.056
Ding 2020 [25]	6 months	I: 80.2%; C: 62.36% HR: 1.18; *p* = 0.49	N/A	I: 16.48%; C: 8.6% HR: 1.98; *p* = 0.24
Eberly 2024 [26]	1 month	N/A	N/A	OR: 0.30 (0.11–0.85) *p* = 0.02
Galinier 2020 [28]	18 months	RR: 0.97 (0.78–1.21) *p* = 0.77	N/A	RR: 0.84 (0.62–1.15) *p* = 0.28
Giordano 2007 [29]	12 months	RR: 0.57 (0.39–0.84) *p* = 0.03	RR: 0.56 (0.38–0.82) *p* = 0.003	RR: 0.49 (0.31–0.76) *p* = 0.0001
Koehler 2010 [30]	24 months	RR: 0.96 (0.83–1.12) *p* = 0.61	N/A	RR: 0.94 (0.72–1.22) *p* = 0.65
Koehler 2011 [31]	26 months	HR: 1.12 (0.91–1.37) *p* = 0.29	HR: 1.07 (0.84–1.35) *p* = 0.58	HR: 0.84 (0.60–1.18) *p* = 0.32
Kotooka 2018 ^b^ [34]	12 months	HR: (0.479–1.320) *p* = 0.376	HR: (0.171–2.074) *p* = 0.415	HR: (0.534–1.897) *p* = 0.983
Lyngå 2012 [35]	12 months	HR: 0.83 (0.61–1.13) *p* = 0.24	HR: 0.9 (0.65–1.26) *p* = 0.54	N/A
Olivari 2018 ^b^ [37]	12 months	MD: −0.02 (−0.5, –0.4) *p* = 0.91	N/A	MD: −0.1 (−0.5,−0.2) *p* = 0.39
Ong 2016 [38]	6 months	I: 50.8% C: 49.2% *p* = 0.39	N/A	N/A
Pekmezaris 2019 [39]	3 months	N/A	RR: 1.32 (0.52–3.4) *p* = 0.56	RR: 1.27 (0.44–3.6) *p* = 0.65
Shara 2022 [40]	3 months	N/A	N/A	I: 20%; C: 0% *p* = 0.021
Seto 2012 [42]	6 months	RR: 1.03 (0.85–1.25) *p* = 0.51	N/A	N/A
Soran 2008 ^b^ [43]	6 months	HR: 1.09 (0.77–1.53) *p* = 0.62	N/A	HR: 0.71 (0.43–1.17) *p* = 0.18
Victoria-Castro 2024 ^e^ [44]	3 months	I1: 34.8%; C: 25% *p* = 0.93 I2: 36.9; C: 25% *p* = 0.97 I3: 30.4%; C: 25% *p* > 0.99	N/A	I1: 13%; C: 9.1% *p* = 0.64 I2: 6.5%; C: 9.1% *p* = 0.94 I3: 4.4%; C: 9.1% *p* = 0.75
Vuorinen 2014 [45]	6 months	N/A	IRR: 0.812 (0.525–1.256) *p* = 0.351	N/A
Wade 2011 [46]	6 months	RR: 0.93 (0.79–1.09) *p* = 0.51	N/A	N/A
Wagenaar 2019 ^e^ [47]	12 months	I1: HR: 0.98 (0.70–1.38) I2: HR: 0.85 (0.59–1.21)	N/A	I1: HR: 0.65 (0.27–1.60) I2: HR: 0.57 (0.23–1.45)
Weintraub 2010 [48]	90 days	RR: 0.50 (0.25–0.99) *p* = 0.05	N/A	Age < 72 years RR: 0.79 (0.34–1.83) *p* = 0.59 Age > 72 years RR: 0.15 (0.04–0.52) *p* < 0.01
(**c**)				
	**QoL Instrument**	**Follow Up Period**	**Outcome**	***p*-Value**
Balk 2008 [19]	MLwHF (Dutch version)	537 days	No improvement	0.61
	SF-36	537 days	No improvement	Not stated
Dar 2009 [23]	EQ 5D	6 months	No improvement	0.50
	MLwHF	6 months	No improvement	0.60
Koehler 2021 [33]	MCS	12 months	Significant improvement	0.024
	PCS	12 months	Significant improvement	0.011
Mizukawa 2019 [36]	MLwHF	24 months	Significant improvement	0.016
Olivari 2018 [37]	MCS	12 months	Significant improvement	0.04
	PCS	12 months	Significant improvement	<0.0001
Pekmezaris 2019 [39]	MLwHF	3 months	No improvement	0.50
Seto 2012 [42]	MLwHF	6 months	No significant improvement	0.05
Victoria-Castro 2024 ^a^ [44]	KCCQ QoL	3 months	No significant improvement ^a^	>0.05 ^a^

(**a**) ^a^ Study has composite outcomes refer to Table 3b. ^b^ Median days alive presented for all-cause mortality, all-cause readmission, and heart failure readmission are represented as mean with standard deviation. ^c^ median days alive presented for all-cause mortality ^d^ Three arm study with two intervention groups, I1: website based telemonitoring, I2: E-health based telemonitoring. C: Control; HR: Hazard ratio; I: Intervention; N/A: Not available; RR: Relative risk. (**b**) ^a^ Number of hospital admissions presented for all-cause hospitalizations. ^b^ Study has composite outcomes; refer to Table 3b. ^c^ Median days alive presented for all-cause mortality, ^d^ all-cause readmission, and heart failure readmission are represented as mean with standard deviation. ^e^ Three arm study with two intervention groups, I1: website based telemonitoring, I2: E-health based telemonitoring. C: Control; HR: Hazard ratio; I: Intervention; IRR: Incidence rate ratio; MD: Mean difference; N/A: Not available; RR: Relative risk. (**c**) ^a^ Study has three intervention groups: I1: Bodyport arm, I2: Coversa arm, I3: Noom arm. *p* values were 0.47, 0.99, and 0.92, respectively, for these three interventions compared to usual care. EQ 5D: EuroQol 5-Dimension; KCCQ: Kansas City Cardiomyopathy Questionnaire; MCS: Mental component summary; MLwHF: Minnesota Living with Heart Failure Questionnaire; PCS: Physical Component Summary; QoL: Quality of life; SF-36: Short Form-36.

## Data Availability

The data presented in this study are available in the article.

## Supplementary Materials

The following supporting information can be downloaded at: https://www.mdpi.com/article/10.3390/medicina61071277/s1, Supplementary Table S1: Baseline characteristics of included studies. Supplementary Table S2: NYHA class and medications across studies. Supplementary Table S3: Composite outcomes of studies.
