# Supplementary material for: Non-Invasive Telemonitoring in Heart Failure: A Systematic Review
Source: Medicina (Kaunas). 2025 Jul 15;61(7):1277. doi: 10.3390/medicina61071277 (PMC12299789; doi:10.3390/medicina61071277)
Supplement: Supplementary file 1 [file medicina-61-01277-s001.zip › medicina-3664805-supplementary.pdf]

SUPPLEMENTARY MATERIAL

Contents

Literature search 2

Inclusion and exclusion criteria Inclusion Criteria 2

Supplementary Table S1: Baseline characteristics of included studies 4

Supplementary Table S2: NYHA class and medications across the studies 7

Supplementary Table S3: Composite outcomes of studies 9

Definitions of telemonitoring features 10

Evaluation of study outcomes using study characteristics 12

- Heart Failure Rehospitalization and Data Transmission Frequency 12
- All-cause hospitalization and Clinician Interaction 12
- All-Cause Hospitalization and Data Transmission Method 12
- Cardiovascular Rehospitalization and Data Transmission Method 12
- Cardiovascular Rehospitalization and Weight Presence 12
- Heart Failure Rehospitalization and Periodic Check-Up Frequency 13

Literature search

(("Heart Failure"[MeSH Terms] OR "Heart Failure"[All Fields] OR "cardiac failure"[All Fields] OR ("congest heart fail"[Journal] OR "chf"[All Fields])) AND ("Telemedicine"[MeSH Terms] OR ("Telemedicine"[MeSH Terms] OR "Telemedicine"[All Fields] OR "telecare"[All Fields]) OR ("Telemedicine"[MeSH Terms] OR "Telemedicine"[All Fields] OR ("tele"[All Fields] AND "care"[All Fields]) OR "Tele Care"[All Fields]) OR "Tele Care"[All Fields] OR ("remote consultation"[MeSH Terms] OR ("remote"[All Fields] AND "consultation"[All Fields]) OR "remote consultation"[All Fields] OR "teleconsultation"[All Fields] OR "teleconsultations"[All Fields] OR "teleconsult"[All Fields] OR "teleconsultant"[All Fields] OR "teleconsultants"[All Fields] OR "teleconsultative"[All Fields] OR "teleconsulting"[All Fields] OR "teleconsults"[All Fields]) OR ("telehealth s"[All Fields] OR "Telemedicine"[MeSH Terms] OR "Telemedicine"[All Fields] OR "telehealth"[All Fields]) OR ("telemedical"[All Fields] OR "telemedically"[All Fields]) OR "telemonitor*"[All Fields] OR "tele referral"[All Fields] OR "tele referral"[All Fields] OR "Tele-Referrals"[All Fields] OR "medicine virtual"[All Fields] OR "Virtual Medicine"[All Fields] OR ("Telemedicine"[MeSH Terms] OR "Telemedicine"[All Fields] OR "ehealth"[All Fields]) OR "electronic health"[All Fields] OR "Digital health"[MeSH Terms] OR "Digital health"[All Fields] OR ("mhealth s"[All Fields] OR "Telemedicine"[MeSH Terms] OR "Telemedicine"[All Fields] OR "mhealth"[All Fields]) OR "Mobile Health"[All Fields] OR "health mobile"[All Fields] OR "Healthapplication"[All Fields] OR "Health Apps"[All Fields] OR "remote monitor*"[All Fields] OR"remote consultation"[MeSH Terms] OR "Cell Phone"[MeSH Terms] OR ("phone s"[All Fields] OR "phoned"[All Fields] OR "phones"[All Fields] OR "phoning"[All Fields] OR"telephone"[MeSH Terms] OR "telephone"[All Fields] OR "phone"[All Fields]) OR"phone*"[All Fields] OR ("phone s"[All Fields] OR "phoned"[All Fields] OR "phones & quot;[AllFields] OR "phoning"[All Fields] OR "telephone"[MeSH Terms] OR "telephone"[All Fields] OR "phone"[All Fields]))) AND ((randomizedcontrolledtrial[Filter]) AND (2004:2025[pdat]))

<u>Inclusion and exclusion criteria</u>

Inclusion Criteria

- Studies involving patients aged ≥ 18 years diagnosed with New York Heart Association (NYHA) functional class I-IV heart failure. (no restriction to HFpEF, HFmrEF or HFrEF)
- Studies involving a specific subset of the HF population (e.g. patients with LVAD, AF only, specific combination with comorbid conditions)
- Studies involving non-invasive home telemonitoring systems.
- Randomized controlled trials (RCTs) with ≥ 50 sample size.
- Studies included a control group receiving the usual treatment and a clearly defined randomization process.

- Full-text articles published and available
- Studies published from 2004 to 2024.
- Studies published in the English language.
- Research focusing on non-invasive interventions targeting heart failure patients, with or without coexisting conditions.

Exclusion Criteria

- Conference abstracts, design reviews, systematic reviews, or meta-analyses.
- Studies conducted in inpatient, rehabilitation centers, or nursing home settings.
- Research lacking the use of remote or wireless data transmission methods.
- Studies exclusively focus on heart failure telerehabilitation.
- Interventions that were not explicitly designed to target heart failure management.
- eHealth or mHealth studies focus solely on educational training without incorporating patient monitoring.
- Studies involving invasive heart failure telemonitoring techniques.
- Studies that involve pedometer-based exercise interventions.
- Studies that do not include a usual care group as a comparator arm.
- RCTs that describe the study protocol but do not report results.
- RCTs that do not incorporate an alert system.

Studies primarily focused on algorithm development for predictive modeling.

Supplementary Table S1: Baseline characteristics of included studies

First Author and year	Country	Total Population (N)	Intervention (%)	Control (%)	Mean age ± SD	Female %	Whites (%)	Blacks (%)	Hispanics (%)	Others (%)
Achury-Saldaña 2024	Colombia	140	50	50	I: 64.1±12<br>C: 69.4 ±11.2<br>T: 66.75 ±11.6	I: 30.0<br>C:27.2<br>T:28.6	N/A	N/A	N/A	N/A
Asch 2022	USA	552	49.3	50.70	I :64.86 ± 12.39<br>C:64.23 ±11.21<br>T: N/A	I: 51.1<br>C: 43.9<br>T: 47.5	I: 41.2<br>C: 42.5<br>T: 41.8	I: 55.5<br>C:50.0<br>T: 52.7	I: 2.8<br>C: 3.2<br>T: 2.9	I: 3.3<br>C: 7.7<br>T: 5.4
Balk 2008	Netherland	214	47	52.80	I: 65 [a]<br>C:68<br>T:66	I: 36.0<br>C: 25.0<br>T: 30.5	N/A	N/A	N/A	N/A
Blum 2014	USA	206	51	49	I: 72 ± 10<br>C:73 ± 8<br>T: N/A	I: 28.0<br>C: 30.0<br>T: 29.0	I: 57.0<br>C:54.0<br>T:.55.8	I: 44.0<br>C:46.0<br>T: 44.8	N/A	N/A
Chaudhry 2010	USA	1653	50	50	I:61<br>C:61<br>T:61	I: 43.5<br>C: 40.6<br>T: 42.0	I: 43.5<br>C: 40.6<br>T: 42.0	I: 38.0<br>C: 39.9<br>T: 39.0	I: 2.7<br>C: 2.8<br>T: 2.7	I:11.9<br>C:11.6<br>T: 11.7
Cleland 2005[b]	UK, Germany Netherlands	426	40.61	19.95	I: 67 ±13<br>C: 68 ±10<br>T: N/A	I: 20.0<br>C:18.0<br>T: 22.0	N/A	N/A	N/A	N/A
Dar 2009	UK	182	50	50	I: 70 ±12.8<br>C:72 ±10.4<br>T: 72 ±12	I: 32.0<br>C: 35.0<br>T: 33.6	N/A	N/A	N/A	N/A
Dendale 2012	Belgium	160			I:75.9 ± 9.6<br>C:75.6 ± 9.8<br>T: 76 ±10	I: 38.0<br>C:32.0<br>T: 35.0	N/A	N/A	N/A	N/A
Ding 2020	Australia	184	49.40	50.50	I:69.5<br>C:70.8<br>T:70.15	I:27<br>C:19<br>T:23	N/A	N/A	N/A	N/A
Eberly 2024[c]	USA	103	N/A	N/A	N/A	N/A	N/A	N/A	N/A	N/A
Frederix 2018	Belgium	160	50	50	I: 76<br>C: 76<br>T: 76	I: 36<br>C: 33<br>T: 34.5	N/A	N/A	N/A	N/A
Galinier 2020	France	900	56.30	43.70	I:70<br>C:69.7<br>T:42	I:N/A<br>C:N/A<br>T:27.70	N/A	N/A	N/A	N/A
Giordano 2007	Italy	460	50	50	I: 58±10<br>C:56±10<br>T:57±10	I: 16<br>C:14<br>T:15	N/A	N/A	N/A	N/A

Koehler 2010	Germany	710	50	50	I: 66.6<br>C: 66.3<br>T: N/A	I: 17.5<br>C:19.5	N/A	N/A	N/A	N/A
Koehler 2011	Germany	710	50.10	49.90	I:66.9±10.8<br>C:66.9 ±10.5<br>T:	I:19.5<br>C:18<br>T: 18.7	N/A	N/A	N/A	N/A
Koehler 2018	Germany	1571	49	51	I:70<br>C:70<br>T:70	I: 30.3<br>C:30.5<br>T: 30.4	N/A	N/A	N/A	N/A
Koehler 2021	Germany	674	N/A	N/A	N/A	N/A	N/A	N/A	N/A	N/A
Kotooka 2018	Japan	181	49.72	50.27	I:67.1±12.8<br>C:65.4±15.6<br>T:66.25±14.2	I:43.33<br>C:38.88<br>T:41.1	N/A	N/A	N/A	N/A
Lyngå 2012	Sweden	344	53	47	I: N/A<br>C:N/A<br>T: 73	I :N/A<br>C:N/A<br>T:25	N/A	N/A	N/A	N/A
Mizukawa 2019	Japan	59	33.90	32.2	I: 70.5±13.3<br>C: 74.5±12.1	I: 50.0<br>C: 47.3<br>T: 42.7	N/A	N/A	N/A	N/A
Olivari 2018	Italy	339	67.55	32.44	I: 79.6±6.8<br>C: 80.9±7.3<br>T:<br>80.28±7.05	I: 38.9<br>C:34.6<br>T: 36.75	N/A	N/A	N/A	N/A
Ong 2016	USA	1437	49.70	50.20	I: 73<br>C:74	I: 46.6<br>C:47.1<br>T:	I: 54.7<br>C: 54.3<br>T:N/A	I:21.5<br>C:22.7<br>T:N/A	I: 12<br>C:10.9<br>T:N/A	I: 11.8<br>C: 12.1<br>T: N/A
Pekmezaris 2019	USA	104	44	56	I: 58.4<br>C:61.1<br>T:59.9	I:43<br>C:40<br>T:41	N/A	I:67<br>C:71<br>T:69	N/A	N/A
Shara 2022[d]	USA	60	50	50	I:54.45<br>±13.02<br>C: 52.93<br>±12.16<br>T: 54	I:35<br>C:50<br>T: 45	I:25<br>C:10<br>T: 16.6	I:75<br>C: 83.3<br>T: 78.3	I: 0<br>C:3.3<br>T: 1.67	I:0<br>C:3.3<br>T: 3.33
Scherr 2009	Austria	120	50	50	I:N/A<br>C:N/A<br>T: 66	I:25.9<br>C:27.8<br>T:27	N/A	N/A	N/A	N/A
Seto 2012	Canada	100	50	50	I:55. ± 13.7<br>C: 52.3 ±13.7<br>T:N/A	I: 18<br>C: 24<br>T	I: 78<br>C:66<br>T:	I: 10<br>C:8<br>T:	N/A	I: 8<br>C:16<br>T:
Soran 2008	USA	315	57.80	49.20	I:76.9±7.1<br>C:76±6.8<br>T: N/A	I:68.7<br>C:60.6<br>T:64.65	I:57.5<br>C:47.7<br>T:52.6	I:41.9<br>C:52.3<br>T:47.1	I:27.5<br>C:28.4<br>T:27.95	I:0.6<br>C:0.0<br>T:0.3
Victoria-Castro 2024[e]	USA	182	75.8	24.2	I1:59 [a]<br>I2:62.5<br>I3:62<br>C:59<br>T:60.5	I1:19<br>I2:16<br>I3:21<br>C:13<br>T:37.9	I1:65.2<br>I2:54.3<br>I3:58.7<br>C:54.5<br>T:58.2	I1:26.1<br>I2:37<br>I3:39.1<br>C:31.8<br>T:33.5	I1:8.7<br>I2:8.7<br>I3:2.2<br>C:11.4<br>T:7.7	I1:0<br>I2:0<br>I3:0<br>C:2.3<br>T:0.5
Vuorinen 2014	USA	94	50	50	I:58.3<br>C:57.9<br>T:58.1	I:61<br>C:61<br>T:61	N/A	N/A	N/A	N/A
Wade2011	USA	316	50.3	49.7	I:75.8<br>C:78.1	N/A	N/A	I:24.4<br>C:20.4	N/A	N/A

					T: N/A			T: N/A		
Wagenaar 2019[f]	Netherland	450	50	50	I1: 66.7 ± 10.4<br>I2: 66.6 ± 11<br>C: 66.9±11.6	I1: 24.7<br>I2: 25.3<br>C: 27.3	N/A	N/A	N/A	
Weintraub 2010	USA	188	50.5	49.5	I:69.5<br>C:68.5<br>T:69	I:36.8<br>C:31.2<br>T:34	I:85.3<br>C:87.1<br>T:86.2	I:1.5<br>C:5.4<br>T:8	I:3.2<br>C:6.5<br>T:4.8	I:1.1<br>C:1.1<br>T:1.1

[a] Median age present instead of mean; [b]Three-arm study with one intervention group; [c] Cross-over study; [d] Intervention group was per protocol; [e] Study has three intervention groups; I1,Bodyport arm ; I2, Coversa arm ;I3, Noom arm; [f] Three arm study with 2 intervention groups, I1: website based telemonitoring, I2: E-health based telemonitoring I: Intervention C: Control T: Total N/A: Not available

Supplementary Table S2: NYHA class and medications across the studies

First Author and year	Class I %	Class II %	Class III %	Class IV %	RAAS blockade ARNI, ACE-I or ARB %	BB %	MRA %	SGLT2i %	Diuretics %
Achury-Saldaña 2024	I: 45.71<br>C:12.85<br>T:29.28	I: 44.28<br>C: 65.71<br>T: 55.9	I: 5.71<br>C:18.57<br>T:12.14	N/A	N/A	N/A	N/A	N/A	N/A
Asch 2022	N/A	N/A	N/A	N/A	N/A	N/A	N/A	N/A	N/A
Balk 2008	I: 6<br>C: 7<br>T: 6.5	I: 41<br>C: 38<br>T:39.5	I:48<br>C:48<br>T:48	I:2,<br>C:3<br>T:2.5	I:92<br>C:94<br>T:93	I:75<br>C:80<br>T:77.5	I:45<br>C:48<br>T:46.5	N/A	N/A
Blum 2014	N/A	N/A	N/A	N/A	N/A	N/A	N/A	N/A	N/A
Chaudhry 2010	I:5.8<br>C: 6.3<br>T: 6.0	I:36.4<br>C: 37.0<br>T: 36.7	I:50.4<br>C: 51.1<br>T: 50.8	I:7.4<br>C: 5.6<br>T: 6.5	I:66.5<br>C: 67.4<br>T: 66.9	I:80.9<br>C:77.5<br>T:79.2	I:32.2<br>C: 33.5<br>T: 32.8	N/A	Loop diuretic<br>I: 78.2<br>C:78.1<br>T:78.2
Cleland 2005	I:22<br>C: 18<br>T:19.33	I:46<br>C:36<br>T:42	I:23<br>C:42<br>T: 31.66	I:8<br>C: 4<br>T: 7	ACEI only:<br>I: 94<br>C:91<br>T: 94<br><br>ARB only:<br>I: 25<br>C: 50<br>T: 42.7	I: 86<br>C:77<br>T:80.66	I: 80<br>C: 74<br>T: 78	N/A	N/A
Dar 2009	N/A	N/A	N/A	N/A	I:82<br>C: 93<br>T: 71.4	I:57<br>C: 55<br>T: 56	I:43<br>C:38<br>T: 40.7		loop diuretic<br>I:89<br>C:97<br>T:92.9
Dendale 2012	N/A	N/A	N/A	N/A	N/A	N/A	N/A	N/A	N/A
Ding 2020	N/A	I:0.5<br>C:0.6<br>T:0.55	N/A	N/A	I:76<br>C:79<br>T:77.5	I:84<br>C:91<br>T:87.5	I:55<br>C:60<br>T:57.5	N/A	N/A
Eberly 2024	N/A	N/A	N/A	N/A	N/A	N/A	N/A	N/A	N/A
Frederix 2018	N/A	N/A	I:3.1<br>C:3<br>T:0.5		I:95<br>C:88<br>T:91.5		I:43<br>C:42<br>T:42.5		Loop diuretics<br>I:66<br>C:79<br>T-72.5<br><br>Thiazide diuretics<br>I:5<br>C:9<br>T:7
Galinier 2020	I: 6.1<br>C: 7.1<br>T:6.6	I:44.2<br>C:43.4<br>T:43.8	I:38.3<br>C: 40.9<br>T:39.6	I:11.4<br>C:8.6<br>T:10	I:73.4<br>C:75.8<br>T:74.6	I:78.6<br>C:80.7<br>T:79.6	I:45.6<br>C:46.4<br>T:46	N/A	N/A
Giordano 2007	N/A	I: 54<br>C:65<br>T:59.5	N/A	N/A	I:95<br>C:94<br>T:93	I:85<br>C:60<br>T: 72.5	I:69<br>C:55<br>T:62	N/A	I:93<br>C:94<br>T:93.5

Koehler 2010	N/A	N/A	N/A	N/A	I:84<br>C:85<br>T: N/A	I:75<br>C:74<br>T: N/A	I:40<br>C:39<br>T: N/A	N/A	N/A
Koehler 2011	N/A	I:49.7<br>C: 50.6<br>T: 50.1	I:50.3<br>C: 49.4<br>T: 49.9		I:96.6<br>C: 94.1<br>T: 95.4	I:92.1<br>C: 93.0<br>T: 92.5	I: 65.3<br>C: 63.2<br>T: 64.2	N/A	I: 93.8<br>C: 93.5<br>T: 93.7
Koehler 2018	I: 0.4<br>C:1<br>T:0.7	I:52.3<br>C:51.2<br>T:51.8	I:46.9<br>C:47.5<br>T:47.2	I:0.4<br>C:0.3<br>T:0.3	I:82<br>C:83<br>T:82.5	I:92<br>C:92<br>T:91	I:58<br>C:52<br>T:55	N/A	I: 93.7<br>C:93.3<br>T:93.5
Koehler 2021	N/A	N/A	N/A	N/A	N/A	N/A	N/A	N/A	N/A
Kotooka 2018	N/A	I: 77.78<br>C:79.12<br>T:78.45	I: 22.22<br>C:20.88<br>T:21.55		ACEI:<br>I:54.4<br>C:56<br>T:55.2<br><br>ARB:<br>I:35.6<br>C:34.1<br>T:34.85	I:92.2<br>C:86.8<br>T:89.5	I:58.9<br>C:63.7<br>T: 61.3	N/A	I:87.8<br>C:84.6<br>T:86.2
Lyngå 2012	N/A	N/A	I: 96.4<br>C:97.4<br>T: 96.9	I:3.6<br>C:2.6<br>T:3.1	I: 93.4<br>C:99.3<br>T:96.2	I: 93.4<br>C:91.5<br>T: 92.5	I: 43.4<br>C:41.8<br>T:42.6	N/A	N/A
Mizukawa 2019	N/A	N/A	N/A	N/A	I: 70<br>C:78.9<br>T:64.4	I:80<br>C: 84.2<br>T:74.6	N/A	N/A	I:100<br>C:100<br>T: 100
Olivari 2018	N/A	I: 48<br>C:48.2<br>T:48.1	I:47.2<br>C:46.4<br>T:46.8	I:4.8<br>C:5.4<br>T: 5.1	ACEI:<br>I:54.6<br>C:50.9<br>T:52.75<br><br>ARBS:<br>I:15.7<br>C:17.3<br>T: 16.5	I: 68.6<br>C:61.8<br>T: 65.2	I:55<br>C:50<br>T: 52.5	N/A	I:98.7<br>C:93.6<br>T:96.15
Ong 2016	I:0.2<br>C:0.7<br>T:N/A	I:23.4<br>C:25.8<br>T: N/A	I:65.6<br>C:63.9<br>T: N/A	I:10.8<br>C: 9.6<br>T: N/A	I:56.6<br>C:54.6<br>T: N/A	I:73.2<br>C:76.1<br>T: N/A	I: 18.9<br>C:19.7<br>T: N/A	N/A	N/A
Pekmezaris 2019	N/A	I: 28<br>C:31<br>T:30	I:72<br>C:69<br>T:70	N/A	N/A	N/A	N/A	N/A	N/A
Shara 2022	N/A	N/A	N/A	N/A	N/A	N/A	N/A	N/A	N/A
Scherr 2009	N/A	I:13<br>C:13<br>T:13	I:68.5<br>C:61<br>T:64.8	I:18.5<br>C:26.0<br>T:22.2	I:100<br>C:100<br>T: 100	I:78<br>C:87<br>T:85.6	I:43<br>C:39<br>T:40.7	N/A	N/A
Seto 2012	N/A	N/A	I: 42<br>C: 42<br>T: 42	I: 4<br>C:4<br>T:4	I:49<br>C:48<br>T:48.5	I:49<br>C:49<br>T:49	I:23<br>C:29<br>T:26	N/A	I:47<br>C:45<br>T:46
Soran 2008	N/A	I:57.5<br>C:59.3<br>T:58.4	I:42.5<br>C:40.7<br>T:41.6	N/A	I:76.2<br>C:76.8<br>T:76.5	I:81.9<br>C:78.7<br>T:75.3	I:20.6<br>C:21.3<br>T:20.95	N/A	I:93.7<br>C:94.8<br>T:94.25
Victoria-Castro 2024	I1:4.3<br>I2:8.7<br>I3:4.3<br>C:3.8	I1:26.1<br>I2:28.3<br>I3:26.1<br>C:38.6	I1:39.6<br>I2:47.8<br>I3:65.2<br>C:43.2	N/A	I1:76.1<br>I2:71.7<br>I3:60.9<br>C:77.3	I1:87<br>I2:91.3<br>I3:89.1<br>C:93.2	I1:47.8<br>I2:45.7<br>I3:56<br>C:52.3	I1:21.7<br>I2:30.4<br>I3:32.6<br>C:31.8	N/A

	T:6	T:29.7	T:56.6		T:71.4	T:90.1	T:50.5	T:29.1	
Vuorinen 2014	N/A	I: 40<br>C:36<br>T:38	I:58<br>C:60<br>T:59	I:2<br>C:4<br>T:3	N/A	N/A	N/A	N/A	N/A
Wade2011	N/A	N/A	N/A	N/A	I: 31.1<br>C:20.4<br>T: N/A	N/A	N/A	N/A	N/A
Wagenaar 2019	I1:57<br>I2:69<br>C:57	I1: 36.8<br>I2: 32.6<br>C: 38.5	I1:11.8<br>12:12.1<br>C:16.8	I1:11.8<br>I2: 6.4<br>C: 4.9	I1:76.7<br>I2:76.7<br>C:81.3	I1: 82.0<br>I2: 80.7<br>C: 85.3	I1: 44.0<br>I2: 39.3<br>C: 40.7	N/A	I1:76.7<br>I2: 66.7<br>C: 80.7
Weintraub 2010	I:1.1<br>C:2.2<br>T:1.6	I:44.2<br>C:47.3<br>T:45.7	I:50.5<br>C:49.5<br>T:50	I:4.2<br>C:1.1<br>T:2.7	I:88.4<br>C:82.8<br>T:32.6	I:92.6<br>C:83.9<br>T:33.6	I:20<br>C:21.5<br>T:7.9	N/A	N/A

Note: ACEi, angiotensin-converting enzyme inhibitor; ARB, angiotensin ii receptor blocker; ARNI, angiotensin receptor-neprilysin inhibitor; BB, beta-blocker; C, control; I, intervention; MRA, mineralocorticoid receptor antagonist; N/A, not available; RAAS, renin angiotensin aldosterone system; SGLT2i, sodium-glucose cotransporter-2 inhibitor; T, total.

Supplementary Table S3: Composite outcomes of studies

Composite Outcomes	All-cause mortality or All-cause readmission	All-cause mortality and rehospitalization for heart failure	Cardiac death or heart failure hospitalization	Cardiovascular mortality or re-hospitalization	Readmission or observation or mortality	Cardiovascular readmission or mortality
Asch 2022	N/A	N/A	N/A	HR<br>0.90<br>(0.72-1.12)<br>p = 0.34	HR:<br>0.88<br>(0.71-1.11)<br>p = 0.26	HR:<br>0.93<br>(0.73-1.19)<br>p= 0.56
Chaudhry 2010	HR<br>1.04<br>(0.91 - 1.19)<br>p = 0.58	N/A	N/A	N/A	N/A	N/A
Kotooka 2013	N/A	HR<br>(0.548-1.648)<br>p = 0.572	N/A	N/A	N/A	N/A
Mizukawa 2019	N/A	N/A	N/A	N/A	N/A	I vs C<br>p = 0.068
Olivari 2018	N/A	RR	N/A	N/A	N/A	N/A

		0.95<br>(0.74-1.22)<br>p = 0.78				
Scherr 2009	N/A	N/A	N/A	RR:<br>0.5<br>(0.3-0.74)<br>p= 0.06	N/A	N/A
Soran 2008	HR:<br>1.04<br>(0.74 - 1.44)<br>p = 0.88	N/A	HR:<br>0.66<br>(0.442 - 1.05)<br>p = 0.083	N/A	N/A	N/A

C: control HR: hazard ratio I: Intervention N/A: not available RR: relative risk

Definitions of telemonitoring features

Term	Definition
Reactive	The care team responds to patient data only after a concerning event or abnormal value is detected, typically addressing issues as they arise.
Proactive	The care team regularly reviews data and engages with patients to prevent issues, offering guidance or adjustments before problems develop
Periodic	Patient data is reviewed and feedback is provided at scheduled intervals, regardless of real-time changes
Continuous feedback	Data is monitored in real time or near real time, enabling immediate feedback and timely interventions based on ongoing data flow
Device-to-provider direct	Data is sent in real time or near real time directly from the patient's device to the healthcare provider's system without intermediaries.

Cloud-based	Data is transmitted to a secure cloud platform, where providers can access it anytime, allowing flexible review and integration.
Stored locally and uploaded periodically	Data is stored on the device and uploaded manually or at scheduled times, rather than in real time
Passive data collection	Occurs when the patient does not actively engage with the device, but the device automatically records the data requested and sends it to the team
Active engagement	Patients manually record data such as weight and blood pressure and submit it themselves
Event-based monitoring	Data is captured or alerts are triggered only when a predefined clinical threshold or event occurs
Routine review of data	Health data is reviewed at regular intervals regardless of alerts or events.
Regular interaction with the patient	The care team engages with the patient consistently through scheduled communication or check-ins.
Short-term	<3 months
Medium-term -	3-6 months
Long-term	> 6 months
Standalone system	A self-contained device or software that functions independently without requiring integration with other systems
Linked to EHRs	A system that connects with Electronic Health Records to automatically share and store patient data within clinical workflows
Community care integration	The coordination of medical services with community-based support to provide comprehensive, patient-centered care.

Evaluation of study outcomes using study characteristics

Heart Failure Rehospitalization and Data Transmission Frequency

Among 15 studies, 4 were not rehospitalized, while 11 were. Studies that avoided rehospitalization had a transmission frequency of weekly (2 studies) or daily (1 study), while none with monthly or every-3-months transmission avoided rehospitalization. In contrast, 9 of 11 rehospitalized studies (82%) had daily transmission. A significant association was found (Pearson $Chi^2(4) = 10.40$, $p = 0.034$; Fisher's Exact Test, $p = 0.033$). The strongest contributions came from studies with daily transmission and rehospitalization (0.4) and weekly transmission with no rehospitalization (4.0).

All-cause hospitalization and Clinician Interaction

Among 18 studies, 2 avoided hospitalization, both with high clinician interaction (active and passive). Of the 16 hospitalized studies, 12 (75%) had passive clinician interaction, while 4 (25%) had proactive interaction. None had both active and passive interaction. A strong association was observed (Pearson $Chi^2(2) = 18.000$, $p = 0.000$; Fisher's Exact Test, $p = 0.007$). The highest contribution (14.2) came from studies with high clinician interaction and no hospitalization.

All-Cause Hospitalization and Data Transmission Method

Among 18 studies, 2 avoided hospitalization—1 using device-to-provider transmission and 1 using stored-locally & uploaded-periodically transmission. All 12 studies using cloud-based transmission were hospitalized, as were 4 of 5 using device-to-provider. A significant association was found (Pearson $Chi^2(2) = 9.900$, $p = 0.007$; Fisher's Exact Test, $p = 0.033$). The highest contribution (7.1) came from stored-locally & uploaded-periodically transmission with no hospitalization, suggesting a protective effect.

Cardiovascular Rehospitalization and Data Transmission Method

Among 8 studies, 2 avoided rehospitalization, both using device-to-provider transmission, while all 6 using cloud-based transmission were rehospitalized. A significant association was found (Pearson $Chi^2(1) = 8.000$, $p = 0.005$; Fisher's Exact Test, $p = 0.036$). The strongest contribution (4.5) came from studies using device-to-provider transmission with no rehospitalization, suggesting better outcomes than cloud-based monitoring.

Cardiovascular Rehospitalization and Weight Presence

Among 8 studies, 2 avoided rehospitalization, both with no weight present, while all 6 with weight present were rehospitalized. A significant association was observed (Pearson $Chi^2(1) = 8.000$, $p = 0.005$; Fisher's Exact Test, $p = 0.036$). The strongest contribution (4.5) came from weight absence with no rehospitalization, suggesting weight presence may be a risk factor.

Heart Failure Rehospitalization and Periodic Check-Up Frequency

Among 15 studies, 4 avoided rehospitalization, mostly with weekly (2 studies) or every 3 weeks (1 study) check-ups. No studies with every 3 months or monthly check-ups avoid rehospitalization. Among 11 rehospitalized studies, 9 (82%) had daily check-ups, while 1 had every 3 months, and 1 had monthly check-ups. A significant association was found (Pearson $Chi^2(4) = 10.40$, $p = 0.034$; Fisher's Exact Test, $p = 0.033$). The strongest contributions came from weekly check-ups with no rehospitalization (4.0) and daily check-ups with rehospitalization (0.4). These findings suggest that weekly or every-3-weeks check-ups may reduce rehospitalization rates.
